# Blood transfusion had no influence on the 5-year biochemical recurrence after robot-assisted radical prostatectomy: a retrospective study

**DOI:** 10.1186/s12894-021-00926-0

**Published:** 2021-11-17

**Authors:** Jiwon Han, Young-Tae Jeon, Jung-Hee Ryu, Ah-Young Oh, Hwanik Kim, Yu Kyung Bae, Chang-Hoon Koo

**Affiliations:** 1 Department of Anesthesiology and Pain Medicine, Seoul National University Bundang Hospital, Seongnam, 13620 Korea; 2 Department of Anesthesiology and Pain Medicine, Seoul National University College of Medicine, Seoul, 03080 Korea; 3 Department of Urology, Seoul National University Bundang Hospital, Seongnam, 13620 Korea

**Keywords:** Biochemical recurrence, Red blood cell transfusion, Robot-assisted laparoscopic radical prostatectomy, Prostate cancer

## Abstract

**Background:**

Although red blood cells (RBC) transfusion is known to be significantly associated with biochemical recurrence in patients undergoing open prostatectomy, its influence on biochemical recurrence after robot-assisted laparoscopic radical prostatectomy remains unclear. Therefore, this study aimed to validate the effect of RBC transfusion on the 5-year biochemical recurrence in patients undergoing robot-assisted laparoscopic radical prostatectomy.

**Methods:**

This study retrospectively analyzed the medical records of patients who underwent robot-assisted laparoscopic radical prostatectomy at single tertiary academic hospital between October 2007 and December 2014. Univariate and multivariate Cox proportional hazard regression analysis was performed to identify any potential variables associated with 5-year biochemical recurrence.

**Results:**

A total of 1311 patients were included in the final analysis. Of these, 30 patients (2.3%) were transfused with RBC either during robot-assisted laparoscopic radical prostatectomy or during their hospital stay, which corresponded to 5-year biochemical recurrence of 15.7%. Multivariate Cox proportional hazard regression analysis showed that RBC transfusion had no influence on the 5-year biochemical recurrence. Variables including pathologic T stage (Hazard ratio [HR] 3.5, 95% confidence interval [CI] 2.4–5.1 *p* < 0.001), N stage (HR 2.3, 95% CI 1.5–3.7, *p* < 0.001), Gleason score (HR 2.4, 95% CI 1.8–3.2, *p* < 0.001), and surgical margin (HR 2.0, 95% CI 1.5–2.8, *p* < 0.001) were independently associated with the 5-year biochemical recurrence.

**Conclusions:**

RBC transfusion had no significant influence on the 5-year biochemical recurrence in patients undergoing robot-assisted laparoscopic radical prostatectomy.

## Background

Robot-assisted laparoscopic radical prostatectomy (RARP) is widely used to treat prostate cancer [1]. Advantageously, RARP reduces blood loss, lowers postoperative pain, leads to fewer complications, and promotes better functional outcomes, when compared to conventional approaches [2–4].

One common benchmark for evaluating treatment efficacy is biochemical recurrence (BCR), which is used as a surrogate marker for prostate cancer and prognostic index for cancer progression, metastasis, and prostate specific mortality [5, 6]. Several studies, as well as a recent meta-analysis, reported that BCR-free survival rates were comparable in patients treated with open radical prostatectomy versus RARP [7, 8]. The 5-year BCR is reported to be 17–19% after open radical prostatectomy [5, 9], and about 14% after RARP [10]. Previous work has identified preoperative prostate specific antigen (PSA), pathologic T stage, surgical margin, as independent predictors of 5-year BCR following open prostatectomy [6]. Moreover, a recent meta-analysis showed that blood transfusion increased the 5-year BCR in patients undergoing open prostatectomy [11]. However, it has not yet been established whether blood transfusion can increase the 5-year BCR in patients undergoing RARP. Therefore, this study aimed to evaluate the effect of blood transfusion on the 5-year BCR in patients undergoing RARP.

## Materials and methods

### Ethics statement

This retrospective study was approved by the Institutional Review Board of Seoul National University Bundang Hospital (SNUBH; Approval Number: B-2005/615-105). Given the retrospective design, the need for informed consent was waived.

### Population

We examined the electronic medical records of patients who were diagnosed with prostate cancer and underwent RARP at SNUBH between October 2007 and December 2014. RARP has been performed in our institution using the da Vinci Surgical System since October 2007. We excluded patients who received radiation therapy or hormonal treatment prior to surgery. Patients whose baseline or postoperative PSA data were missing or incomplete, were also excluded. We also excluded patients whose RARP was converted to open surgery.

### Surgical procedures

RARP was conducted by experienced surgeons via transperitoneal approach using the four-armed da Vinci surgical robot system. Patients were placed in lithotomy position. After docking, the following surgical procedures were performed; bladder detachment, endopelvic fascial incision, dissection of dorsal venous complex, bladder neck, vas deference, seminal vesicle, tissue around the prostate, reconstruction and vesicourethral anastomosis [12]. Adjuvant hemostatic agents (mixture of thrombin with gelatin or fibrin) were routinely used by the surgeons. Patients had a Jackson–Pratt drain placed anteriorly to the bladder wall through one of the lateral port sites and secured with a 4-0 nylon stitch. Unless the operation violated oncological guidelines, the neurovascular bundle was spared [13].

### Data collection and outcomes

Blood transfusion data were retrieved from electronic medical records of SNUBH and used to evaluate the efficacy of transfusion. Perioperative transfusion was defined as red blood cells (RBC) transfusion either during RARP or within the postoperative hospital stay. RBC was transfused according to the clinical decision of physicians. The demographic (age and body mass index), clinical, and pathological data of patients were collected as covariates. Collected clinical data included American society of anesthesiologists physical status, smoking history, main anesthetic agents, the administration of intraoperative fluid, estimated blood loss during surgery, duration of surgery and anesthesia, and length of hospital stay. Pathologic evaluation of the specimens was consistently performed by urologic pathologists. Pathologic T stage, N stage, margin status, and Gleason score were collected.

BCR was defined as two consecutive serum PSA level of ≥ 0.2 ng/mL. The initial postoperative PSA was evaluated after 6 weeks. Thereafter, PSA was assessed every 3 months for the first year, and every 6 months during the subsequent 4 years.

### Statistical analysis

Continuous variables were presented as median with interquartile ranges (IQR), categorical variables were presented as number with percentage. Univariate Cox proportional hazard regression analysis was performed to identify any potential variables associated with the 5-year BCR. Subsequently, variables with a *p *value < 0.1 in univariate analysis were selected for multivariate Cox proportional hazard regression analysis using forward selection step. In regression analyses, we categorized the pathologic T stage as < pT3 or ≥ pT3, and the Gleason score as < 8 or ≥ 8. All statistical analyses were performed using SPSS 24. A *p* value < 0.05 was considered statistically significant.

## Results

**Fig. 1:**
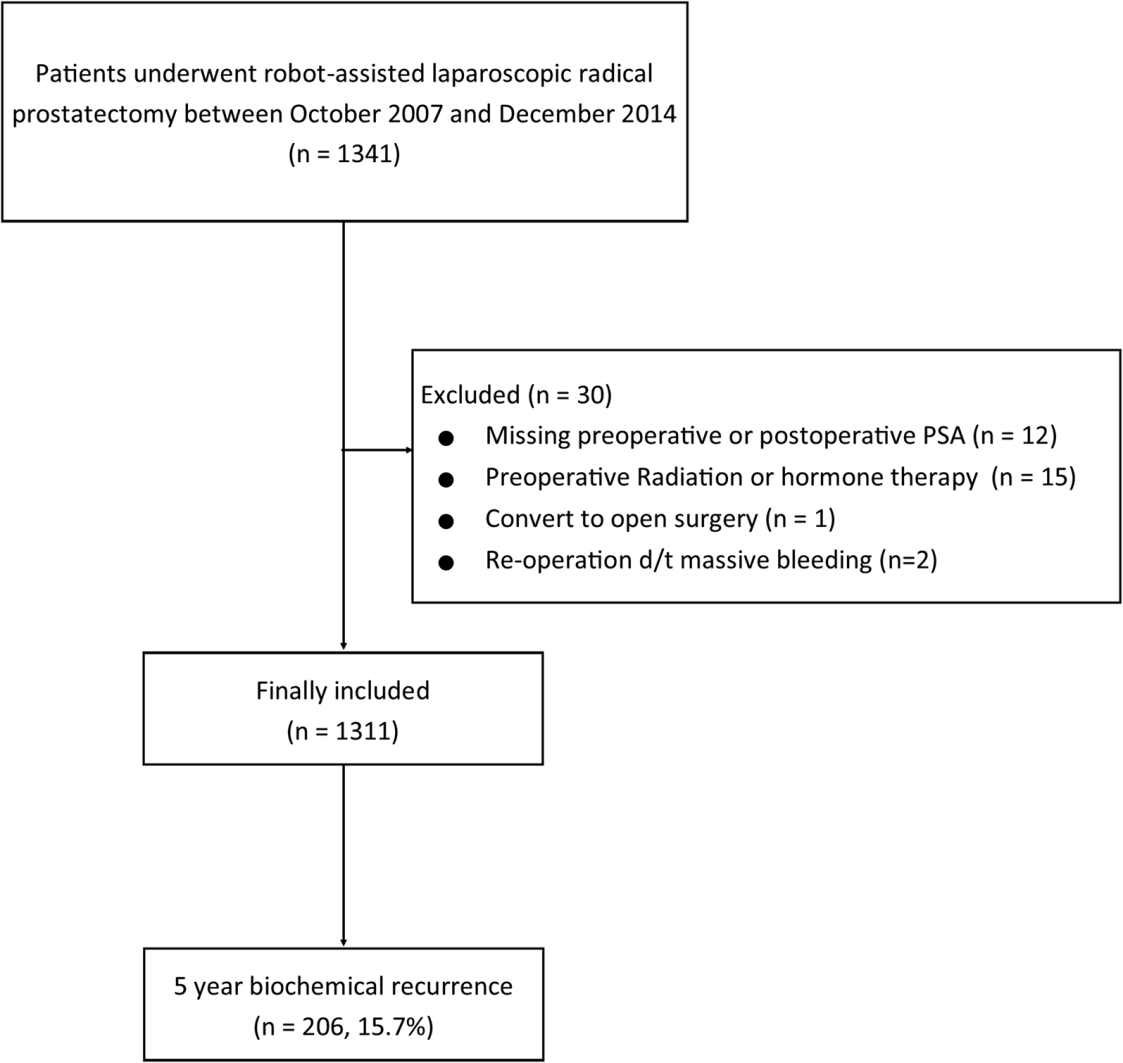
Flow diagram outlining the inclusion and exclusion criteria with assignment of study cohort

A total of 1341 patients underwent RARP between October 2007 and December 2014 at SNUBH. Among them, 12 patients were excluded due to a lack of preoperative or postoperative PSA data. An additional 15 patients were excluded for receiving radiation therapy or hormonal treatment prior to surgery. Finally, three additional patients were excluded because they were converted from RARP to the open technique or they received re-operation before discharge. As such, a total of 1311 patients were included in the final analysis (Fig. 1).

Demographic characteristics are summarized in Table 1, and pathologic characteristics are presented in Table 2. Among 1311 patients, 30 patients (2.3%) were transfused with RBCs during RARP or the hospital stay. The median follow-up period after RARP was 102 [IQR 60–146] months, and the 5-year BCR was 15.7% (n = 206). The median time to BCR was 9.8 [IQR 3.4–30.1] months.

**Table 1:** Clinical characteristics of included patients (n = 1311)

Variable	
*Preoperative variables*	
Age, year	66 [61–71]
BMI, kg/m^2^	24.6 [22.8–26.2]
ASA physical status, n	
I	436 (33.3%)
II	829 (63.2%)
III	46 (3.5%)
Smoker, n	490 (37.4%)
Preoperative PSA, ng/ml	8.9 [5.6–15.8]
Preoperative Hb, g/dl	14.6 [13.8–15.4]
Preoperative Hct, %	42.8 [40.4–44.9]
*Year*	
2007-2008	196 (15.0%)
2009-2010	372 (28.4%)
2011-2012	377 (28.8%)
2013-2014	366 (27.9%)
*Intraoperative variables*	
Main anesthetic agent, n	
Inhalation agent	1217 (92.8%)
Propofol	94 (7.2%)
Fluid administered	
Crystalloid, ml	1204 [900–1651]
Estimated blood loss, ml	200 [100–300]
Duration of surgery, min	200 [175–225]
Duration of anesthesia, min	250 [225–275]
*Perioperative transfusion*	
Patients who were transfused, n	30 (2.3%)
Number of packed RBC in patients transfused	2 [2–3]
*Length of hospital stay, d*	11 [9–12]

Values are expressed as median [interquartile range] or number (percentage)

*BMI* body mass index, *ASA* American society of anesthesiologists, *PSA* prostate-specific antigen, *Hb* hemoglobin, *Hct* hematocrit

**Table 2:** Pathological characteristics of included patients (n = 1311)

Variable	
Pathologic T stage, n	
pT2	3 (0.2%)
pT2a	109 (8.3%)
pT2c	771 (58.8%)
pT3a	291 (22.2%)
pT3b	129 (9.8%)
pT4	8 (0.6%)
Pathologic N stage, n	
Nx	792 (60.4%)
N0	486 (37.0%)
N1	33 (2.5%)
Gleason score, n	
≤ 6	116 (8.8%)
7	1025 (78.2%)
8	62 (4.7%)
9	108 (8.2%)
Surgical margin positive, n	393 (30.0%)
1 year BCR	79 (6.0%)
5 year BCR	206 (15.7%)

Values are expressed as median [interquartile range] or number (percentage)

*BCR* biochemical recurrence

Table 3 shows the results of univariate and multivariate Cox proportional regression analysis for the 5-year BCR. In univariate analysis, RBC transfusion was associated with the 5-year BCR (HR 2.159, 95% CI 1.107–4.211, *p* = 0.024). The result also showed the significant association between the number of transfused RBC and 5-year BCR (HR 1.335, 95% CI 1.094–1.631, *p* = 0.005). However, when they were included in the multivariate analysis with preoperative PSA level, pathologic T stage, N stage, gleason score and surgical margin status, both variables were not statistically significant, respectively (*p* > 0.05). Other variables, including preoperative PSA (HR 1.005, 95% CI 1.002–1.009, *p* < 0.001), pathologic T stage ≥ pT3 (HR 3.510, 95% CI 2.426–5.078, *p* < 0.001), N stage (HR 2.337, 95% CI 1.465–3.729, *p* < 0.001), Gleason score ≥ 8 (HR 2.373, 95% CI 1.756–3.207, *p* < 0.001) and positive surgical margin (HR 2.010, 95% CI 1.452–2.782, *p* < 0.001) were found to be independent predictor of 5-year BCR.

**Table 3:** Univariate and Multivariate Cox proportional hazard regression analysis for 5-year biochemical recurrence of prostate cancer

Variable	Univariate analysis		Multivariate analysis	
	HR (95% CI)	*p* value	HR (95% CI)	*p* value
*Preoperative variables*				
Age, year	1.015 (0.994–1.035)	0.155		
BMI, kg/m2	0.991 (0.951–1.034)	0.678		
ASA physical status				
I	1 (reference)			
II	0.794 (0.597–1.056)	0.112		
III	0.934 (0.451–1.934)	0.854		
Diabetes mellitus	1.321 (0.887–1.968)	0.171		
Hypertension	1.188 (0.887–1.592)	0.248		
Coronary artery disease	1.421 (0.840–2.404)	0.190		
Cerebrovascular accident	1.382 (0.651–2.937)	0.400		
Smoker	0.933 (0.702–1.241)	0.634		
Preoperative PSA, ng/ml	1.015 (1.013–1.018)	< 0.001*	1.005 (1.002–1.009)	< 0.001*
Preoperative Hb, g/dl	0.916 (0.826–1.016)	0.098		
*Intraoperative variables*				
Main anesthetic agent				
Inhalation agent	1 (reference)			
Propofol	0.957 (0.556–1.646)	0.874		
Crystalloid, ml	1.000 (1.000–1.000)	0.968		
Colloid, ml	1.000 (1.000–1.001)	0.034*		
Estimated blood loss, ml	1.001 (1.000–1.001)	0.042*		
*Perioperative RBC transfusion*				
None	1 (reference)			
Transfusion	2.159 (1.107–4.211)	0.024*		
Number of packed RBC	1.335 (1.094–1.631)	0.005*		
*Pathological variables*				
Pathologic T stage				
< pT3	1 (reference)			
≥ pT3	7.197 (5.264–9.839)	< 0.001*	3.510 (2.426–5.078)	< 0.001*
Pathologic N stage				
Nx or N0	1 (reference)			
N1	7.080 (4.504–11.129)	< 0.001*	2.337 (1.465–3.729)	< 0.001*
Gleason score				
< 8	1 (reference)			
≥ 8	5.478 (4.137–7.254)	< 0.001*	2.373 (1.756–3.207)	< 0.001*
Surgical margin positive	4.821 (3.628–6.404)	< 0.001*	2.010 (1.452–2.782)	< 0.001*

Statistically significant *p* value (< 0.05) are highlighted in bold

*BMI* body mass index, *ASA* American society of anesthesiologists, *PSA* prostate-specific antigen, *Hb* hemoglobin

## Discussion

This study showed that perioperative transfusion was not independent factor for 5-year BCR in patients undergoing RARP. In addition, this study found that pathologic T stage, N stage, Gleason score, and surgical margin were independently associated with an increased 5-year BCR.

Several studies focused on how transfusion causes pro-tumorigenic environment and supported a significant association between transfusion and increased recurrence in patients undergoing surgery for colon, stomach, liver, or bladder cancer [14–17]. There are several pathophysiology to explain the effect of blood transfusion on tumor recurrence in patients undergoing cancer surgery. Surgical manipulation may enable malignant cells to circulate in the bloodstream [18, 19]. Moreover, anesthetics and opioids attenuate host immunity, leading to a permissive tumor environment [20]. Indeed, previous work assessed perioperative changes in the ratio of Th1/Th2 cells period in surgical patients, and observed a shift toward a Th2 immune response, indicative of a significant alteration in the composition of the immune system [21]. Post-surgical immunosuppression may be further aggravated by transfusion as RBCs can also modulate the immune system, a phenomena referred to as transfusion related immunomodulation (TRIM). There are 4 mechanisms for TRIM; (1) suppression of cytotoxic cell and monocyte activity; (2) release of immunosuppressive prostaglandins; (3) inhibition of interleukin-2 production; (4) increased suppressor T-cell activity [22]. Contrary to those studies, we found that transfusion was not associated with increased 5-year BCR in patients undergoing RARP.

There has been some disagreement with regard to whether transfusion may influence on BCR after radical prostatectomy. As mentioned in the introduction, a recent meta-analysis including 8 studies showed that perioperative blood transfusion was associated with decreased overall survival and recurrence-free survival rate following open radical prostatectomy [11]. In contrast to these findings, Boehm et al. analyzed 11,723 patients who were treated with open radical prostatectomy or RARLP and found no correlation between blood transfusion and oncological outcomes [23]. The authors explained this discordance by changes in transfusion standard and reduced immunologic reaction related to transfusion. To the best of our knowledge, this is the first study to evaluate the effect of blood transfusion on BCR in patients undergoing RARP solely.

In the present study, the average patient blood loss was 200 ml, and transfusion rate was 2.3%. These are comparable to previous work which showed that RARP reduced blood loss (188 ml vs. 745 ml) and transfusion rates (16.5% vs. 1.8%) when compared with open prostatectomy [24]. Among the patients who received blood transfusion, the median number of transfused RBC was 2 units. Given that relatively low blood loss and volume, it can be inferred that the volume of transfused RBC was not enough to induce pro-tumor environment or immunomodulation. Difficult surgical conditions such a locally advanced tumor, which is T3 or T4, may increase the risk of transfusion. Therefore, we assessed multicollinearity which means a linear relationship between T stage and transfusion using variance inflation factor. The value of 1.384 suggests that multicollinearity was not a problem in our model.

Consistent with previous work, this study showed that Gleason score, T stage, N stage, and surgical margin, were independent predictors of 5-year BCR. Several large studies with a follow-up more than 5 years have identified predictors of BCR after RARP [10, 25–27]. Despite slightly different definitions of variables among these studies, most studies have reported that Gleason score, pathologic stage, and/or surgical margin were independently associated with 5-year BCR.

It is important to recognize that BCR is not a definitive indicator for the clinical relapse of prostate cancer. However, BCR has been associated with increased mortality [28]. Therefore, physicians should be aware of predictive factors for BCR in prostate cancer patients, and therein stratify high risk patients to prevent clinical progression.

There are several limitations in this study. First, given that relatively small proportion of transfused patient, our findings needed to be interpreted with caution. However, transfusion rate of the present study was similar to the previous study [24]. Second, transfusion was performed in accordance with conventional criteria (Hb 7–8 g/dL or less or Hb 10 g/dL or less if patients has ischemic disease such as coronary atherosclerosis or stroke) but without a predefined protocol. Given that our results are based on a retrospective analysis, further prospective studies are therefore required to validate the association between transfusion and 5-year BCR. Third, the impact of RBC storage was not considered in this study. It has been reported that prolonged RBC storage may have detrimental clinical impacts [29]. However, the small number of patients who were transfused in this study was seemingly insufficient to draw meaningful results from a subgroup analysis based on the duration of RBC storage.

## Conclusions

The evidence from this study suggests that blood transfusion was not associated with an increased 5-year BCR in patients undergoing RARP. We hope that our research will be helpful in terms of perioperative blood management in patients undergoing RARP.

## Data Availability

The datasets generated and analyzed during the current study are available from the corresponding author on reasonable request.
